# Environmental impacts of drugs against parasitic vector-borne diseases and the need to integrate sustainability into their development and use

**DOI:** 10.12688/openreseurope.18008.2

**Published:** 2024-11-04

**Authors:** Clara M. Lima, Elisa Uliassi, Eli S.J. Thoré, Michael G. Bertram, Luis Cardoso, Anabela Cordeiro da Silva, Maria Paola Costi, Harry P. de Koning

**Affiliations:** 1 Host-Parasite Interaction Group, Institute for Research and Innovation in Health, University of Porto, 4200-135, Porto, Portugal; 2 Microbiology Laboratory, Department of Biological Sciences, University of Porto, Porto, 4050-313, Portugal; 3 Department of Pharmacy and Biotechnology, University of Bologna, Bologna, 40126, Italy; 4 Department of Wildlife, Fish, and Environmental Studies, Swedish University of Agricultural Sciences, Umea, SE-907 36, Sweden; 5 Laboratory of Adaptive Biodynamics, Research Unit of Environmental and Evolutionary Biology, Institute of Life, Earth and Environment, University of Namur, Namur 5000, Belgium, Namur, 5000, Belgium; 6 Department of Zoology, Stockholm University, Stockholm, Stockholm 114 18, Sweden; 7 School of Biological Sciences, Monash University, Melbourne, Victoria, 3800, Australia; 8 Department of Veterinary Sciences, and Animal and Veterinary Research Centre, University of Trás-os-Montes e Alto Douro, Vila Real, 5000-801 Vila Real, Portugal; 9 Associate Laboratory for Animal and Veterinary Sciences (AL4AnimalS), Faculdade de Medicina Veterinária, University of Trás-os-Montes e Alto Douro, Lisbon, 5000-801 Vila Real, Portugal; 10 Department of Life Science, University of Modena and Reggio Emilia, Modena, 41125, Italy; 11 School of Infection and Immunity, College of Medical, Veterinary and Life Sciences, University of Glasgow, Glasgow, Scotland, G12 8TA, UK

**Keywords:** Drug discovery, Ecotoxicology, Environmental impact, One Health, Parasitic vector-borne diseases

## Abstract

**Background:**

The current scientific discourse on environmental impacts of veterinary medicines mostly focuses on ectoparasiticides. Meanwhile, the environmental impacts of widely prescribed drugs for the treatment of human and animal parasitic vector-borne diseases (PVBD) remain largely unexplored. There is thus a need for evidence-based information to support guidelines and protocols for sustainable One Health PVBD drug development and use, while promoting greener research practices. Here, we reflect on the potential environmental impacts of PVBD drugs in current use, and the environmental impact of our research practices for developing new antiparasitics.

**Methods:**

We conducted a survey of the membership of the “One Health drugs against parasitic vector borne diseases in Europe and beyond” Cooperation in Science and Technology (COST) Action 21111 (OneHealth *drugs*) to assess the current appreciation of sustainable drug design concepts and the extent to which One Health and sustainability principles are integrated into PVBD drug discovery and development. The survey also explored which human, technical, and funding resources are currently used in Europe and neighbouring countries in PVBD drugs research.

**Results:**

The survey was conducted and analysed by OneHealth *drugs* and garnered 89 respondents, representing a response rate of 66% from 32 countries, predominantly European. 87% of participating collaborators worked in Academia; research groups were small (60% with 1–4 researchers) and mostly consist of few researchers, mostly at early career stages (63% <35 years old). Collaborations were mostly between academics, and 60% collaborated with non-European researchers, while funding was mostly from national governments. Motivation for greener research practices was high but there was as yet low implementation of green strategies or the incorporation of ecotoxicological test in drug development workflows, due to cost and unfamiliarity.

**Conclusions:**

We highlight the need for early-ecotoxicological testing of new drug candidates and suggest best practices as we move towards standardized protocols in developing safe and efficacious PVBD drugs.

## Introduction

Antiparasitic drugs are used to prevent, treat, and cure parasite-borne diseases in humans and animals. The prescription or administration of antiparasitic drugs typically follows the best interest of the patient or target populations, whether human or animal ^1– 3^. Specifically, it aims at eliminating or alleviating clinical symptoms of disease, thereby improving human and/or animal health and performance. To prevent and/or eliminate parasite-borne diseases, prophylactic and therapeutical antiparasitic drugs each play a key role. These include ectoparasiticides (against arthropods), endoparasiticides (against systemic parasites) and endectocides (against both external and internal parasites) ^4^. While the health benefits of (antiparasitic) drugs are undeniable, the use of such compounds may also come at a substantial environmental cost. Specifically, drugs often enter and contaminate natural environments where they can have a wide range of unintended but far-reaching effects on ecosystems – and from there again on humans and domesticated animals ^5– 7^. Accordingly, we now increasingly appreciate the need to develop and use drugs sustainably, safeguarding the health of humans and animals while also protecting natural ecosystems from their potential impacts ^8^. This approach—rooted in the One Health concept by acknowledging the interdependence between healthy people, healthy animals, and healthy environments, was recently identified as a vital strategy towards more sustainable drug development ^9^.

### State of antiparasitic drug discovery and development

Behind each antiparasite drug hides a great research effort, often spanning decades, and it was during the second half of the 20 ^th^ century that research and development for antiparasitic drugs achieved its pinnacle ^10– 12^. Consequently, most antiparasitic drugs currently in use hail from that period, when antiparasite drug discovery was boosted by advances in synthetic organic chemistry and the unmet medical need for interventions against parasitoses with impacts on human and animal health, food production, and the economy. These drugs have indisputably provided considerable improvements in human and animal health, contributed very significantly to extensive reductions in zoonotic parasitic diseases, and allowed the farming industry to intensify animal-based food production. Simultaneously, the worldwide distribution and large-scale application of antiparasite drugs have rewarded the veterinary pharmaceuticals industry with equally healthy profits ^13^.

Partly as a result of antiparasitic drug successes, society has developed a substantial reliance on intense drug-dependent animal farming for food production. With the easy access to effective and cheap antiparasitic drugs, further antiparasitic drug research and development was disincentivized and became unprofitable. Yet, this long-term, complacent *status quo* is currently no longer tenable. Interspecies boundaries are being challenged by the incursion of agricultural activity into wildlife habitats ^14^, anthropomorphized relationships with companion animals, and the challenges posed by the emergence and reemergence of parasitic zoonotic diseases, and especially the global threat of drug-resistance among arthropod vectors, helminths, and protozoan pathogens ^15, 16^. The urgency of this situation is at odds with the levels of funding with which agencies are willing to support research and development for parasite-borne diseases and parasitology in general ^17^.

The precipitous decline in veterinary antiparasitic drug discovery is part of the overall trend of productivity decline in pharmaceuticals research and development ^18^ but may be partially justified by the fact that the veterinary market for antiparasite formulations is quite stable, accounting for ~23% of the global animal health market ^19, 20^. This stability, and a lack of competing new products, has stifled the need for innovation and garnished complacency. However, the revenue obtained from the sales of the currently licensed antiparasite drugs must be reinvested into research and development towards new compounds, because without it antiparasitic drug discovery risks being seen as an unprofitable and nonviable investment ^13, 18^.

Regarding human health, parasitic diseases of zoonotic or anthroponotic origin persist as a major cause of morbidity and mortality ^21^. Yet many of these diseases are classified as neglected, syndemic illnesses associated with poverty. Such status limits the appetite of funding agencies for supporting academic research into new, safer, and more efficacious antiparasitic human drugs, as the outcome of the research will be unlikely to be taken up by the highly profit-oriented drugs industry. Consequently, treatment and public health measures to control such human parasitic diseases continue to depend on outdated drugs with suboptimal activity and safety margins, often producing severe side effects. Moreover, considering that vaccines for human parasitic diseases and adequate vector control measures are almost non-existent, considerable resistance to these drugs has developed, after decades to more than half a century of intensive use ^13, 15– 17^.

Instead of investing in *ab initio* drug discovery, the veterinary pharmaceutical industry has turned to drug repositioning and repurposing for the development of anti-parasite drugs or drug combinations that enable a broad spectrum chemoprophylactic, parasiticidal, or pesticidal coverage of multiple parasite species ^15, 16, 19, 22^. Some of these drug applications offer long-lasting protection (ranging from weeks to a year-round prevention) after a single application ^23– 25^. However, we have increasingly witnessed and reported the development of cross-resistance, sometimes because multiple drugs against parasitic vector-borne diseases (PVBDs) or their vectors share the same mechanisms of action, sometimes because they share the same transporters ^26– 30^. Furthermore, the broad-spectrum activity of many of these drugs, combined with their (inadvertent) release into the environment, leads to a wide range of potentially far-reaching effects on non-target species in natural ecosystems ^19, 31^ with detrimental impacts on insects, aquatic ecosystems and mammalian species other than those of human and veterinary health concern ^4^.

### Environmental impacts

Over the past decades, major concerns have emerged regarding the widespread use of pharmaceuticals and their release into ecosystems. To date, close to 1000 active pharmaceutical ingredients or their transformation products have already been detected in natural environments all around the globe, of which over 700 in the European Union alone ^32^. In this context, veterinary pharmaceuticals, including antiparasite drugs and their metabolites, are no exception ^33^. Environmental pollution with veterinary drugs results from decades of anti-parasite control strategies that often rely on the large-scale use of a very small number of broad-spectrum compounds, used worldwide to protect the health of livestock and companion animals alike ^13, 19^. In veterinary practice both individual and mass drug administration strategies are often prescribed in a non-evidence-based manner, lacking proper diagnosis, follow-up and monitoring, and without considering the broader environmental impact of their use ^4, 33, 34^.

A striking example of the large-scale consequences of the use of insecticidal and antiparasite treatments comes from the environmental impact of macrocyclic lactones including ivermectin, particularly through their insecticidal effects on the ecology of biologically and economically important insect species ^35– 37^ and aquatic organisms ^38^. On the other hand, the insecticidal action of slow-release injectable ivermectin can be used to render the blood of cattle toxic to biting mosquitos and so contribute to malaria control, if mitigating efforts are taken to manage the ecotoxicological effects of the drug ^39^. This example highlights that innovative OneHealth solutions can produce targeted, beneficial ecological interventions. Similarly, the antidepressant amitriptyline, which acts on pre-synaptic serotonin and norepinephrine reuptake transporters that are highly conserved even in invertebrates, affects the feeding behaviour and reproduction of sweet water molluscs even at very low concentrations ^40^ – a clear ecotoxicological hazard affecting an important part of the fresh-water food chain. Yet, fresh-water snails are also intermediate hosts for *Schistosoma* species – major human and animal pathogens in many tropical regions and are combatted by molluscicides. 

While it is increasingly recognised that pharmaceuticals can have profound environmental impacts, there is currently still a severe lack of information regarding the environmental fate and ecological effects of many antiparasite drugs. In this sense, we consider that the environmental impact of drugs applied in the management of human and animal helminthic and protozoan parasitic diseases should not be underestimated. This is particularly true for legacy drugs that have been on the market for an extended period of time and were approved based on regulatory standards that did not strictly consider the environmental impact of drugs. For many protozoan infections only a small number of drugs is available for the management of human and animal disease, each of them considered essential ^41^. Ideally, all should now be assessed for ecotoxicity, and phased out if they fall short of objective standards. However, this would potentially leave important PVBDs without any medications, including the outdated ones from the past decades, pending suitable newer replacements ^4, 17, 41^. From the above, we perceive that this issue is not of simple resolution, and retroactive application of environmental standards will be impossible given the paucity of treatment options in this case. Moreover, without standardisation of assays and agreement on scales and acceptable limits, it remains impossible to compare the ecological impacts of the existing drugs and any newly developed compounds. Therefore, reliable and standardised guidelines and protocols to accurately assess the ecological risks of drugs are urgently needed ^42^.

The detection of anti-parasitic drugs in environmental can be achieved by high-resolution mass spectrometry coupled with chromatography ^43^, which enables the identification of a wide range of chemical contaminants, including anti-parasitic drugs, across various environmental compartments (e.g., water, sediment, air) and biological matrices (e.g., tissue, blood) ^44, 45^. The sensitivity of these techniques has markedly improved, enabling the detection of trace concentrations of chemical contaminants down to picogram per litre levels ^46^. While the high costs of performing such analyses currently still represents a bottleneck ^47^, these techniques can provide crucial data for understanding environmental exposure scenarios.

### Stakeholder engagement and responsibilities

Currently, the lack of knowledge and consensus methods to assess the environmental impact of pharmaceuticals feeds clashing positions and priorities by the various stakeholders, such as environmental associations (e.g. Pesticide Action Network), public health advocates, veterinary practitioners associations (e.g. https://www.veterinaryprescriber.org/), pharmaceutical companies (e.g. NOAH, the National Office of Animal Health, representing the United Kingdom animal medicines industry), and intergovernmental agencies (e.g. the European Medicines Agency, EMA). Both the European Federation of Pharmaceutical Industries and Associations (EFPIA) and the UK have now established a Pharmaceuticals in the Environment (PiE) group to enable discussion and knowledge exchange relating to pharmaceuticals in the environment from human, veterinary, agricultural, and non-agricultural sources ^48, 49^.

So far, the discussion and research into the environmental impacts of pharmaceuticals has largely focused on the environmental fate of insecticidal and ectoparasitic drugs and their toxic effects on non-target organisms. This is understandable as they are often sprayed in large quantities, with the run-off directly flowing into surrounding natural environment. However, that leaves the impacts of other antiparasite drugs still to be determined, and funding for such work has been uncertain at best. While it is clear that much work still needs to be done before we will fully understand and be able to mitigate the ecological risks of pharmaceuticals, many valuable initiatives have already emerged. For instance, the European Scientific Counsel for Companion Animal Parasites (ESCCAP) promotes a risk assessment for the exposure to endo- and ectoparasites before decisions are made on prophylactic treatments, with the aim of reducing unnecessary drug use. Equally, the World Association for the Advancement of Veterinary Parasitology (WAAVP; https://www.waavp.org/) promotes education and research in the field of Veterinary Parasitology, while disseminating guidelines for a responsible use of veterinary antiparasitic drugs. From the antiparasite research and development perspective, the COST Action “One Health drugs against parasitic vector borne diseases in Europe and beyond” (OneHealth *drugs*; https://www.cost.eu/actions/CA21111/) is a consortium of researchers dedicated to improving drug development against PVBDs of humans and animals, through coordination of the discovery of drugs that help control human and veterinary vector-borne infections, adhering to the principles of the optimal profile for all organisms, while reducing the environmental impact of their associated research and the resulting new treatments.

Importantly, the EMA regulation for marketing authorization of veterinary medicinal products has recently been updated to include new environmental vigilance measures. As a result, the process of new veterinary medicinal product marketing authorisations now includes three phases of risk assessment: Phase I defines the routes of the veterinary medicinal products into the environment and their potential for bioaccumulation and persistence; Phase II estimates the toxic potential of the drug and its metabolites at the predicted environmental concentration against the lowest effective concentrations in standard ecotoxicity tests in soil and/or water; Phase III produces the veterinary medicinal product’s environmental impact assessment ^9, 50^.

### One Health framework applied to antiparasite drugs development and application

Improving drug development for PVBD is required to control vector-borne parasitic infections in human and veterinary settings. This is so, not only to keep up with the challenges posed by drug resistance and climate-associated alterations in the vector-borne diseases landscape, but also to overcome the low efficacy and safety profiles, besides the environmental toxicity, associated with the currently available drugs. The challenge is to produce new compounds with exceptional antiparasite profiles, safeguarding an optimal therapy, while reducing the environmental impact of both the new treatments and of the research that leads to their development. Significant improvement in the drug discovery pipeline will be achieved once the new leads and compounds present optimal safety and efficacy on target parasite/host combinations, while preserving the biological integrity of other organisms, through biodegradability and environmental safety, and reducing the environmental burden of their research and development to the possible minimum.

In this sense, a successful and sustainable program for antiparasite drug discovery and delivery should be built on a *One Health* framework, contemplating the mobilization of scientific know-how across different disciplines, promoting operationalization, management, and delivery of knowledge between relevant academic institutions (medicinal chemistry, parasitology, entomology, human and veterinary medicine, ecology, ecotoxicology and conservation) and stakeholders (pharmaceutical industry and policymakers).

The importance of OneHealth in drug development has been made cogently and urgently for antibiotics ^51^ but also beyond ^52^. Humans alone consumed well over 3 trillion doses of pharmaceuticals in 2022 ^53^ and in addition large quantities of drugs are administered to livestock and companion animals: in 2020 the global usage of antimicrobials for cattle, pigs, sheep and chickens alone was estimated to be 99,502 tonnes and projected to increase by a further 8% by 2023 ^54^. At the same time, the number of companion animals is growing, at least in Europe, but there is no EMA requirement to assess the environmental impact of drugs to treat household pets, in contrast to farm animals ^40^.

The combined One Health impact of the production, usage and disposal of all veterinary drugs is potentially huge. A particularly instructive One Health impact of a veterinary drug is the non-steroidal anti-inflammatory drug diclofenac which, given to cattle, had the unintended consequence of poisoning vultures in the Indian subcontinent ^55^, leading to a collapse of the species and, consequently, of the ‘sanitation services’ they provided. Frank and Sudarshan estimate that this in turn caused an increased human mortality rate of 4.7% in some districts ^56^.

## Methods

### Survey methodology

In an attempt to catalogue and harness the current research activities related to PVBD drug development and gain an understanding of the scope for integrating sustainable drug design concepts and One Health principles into this current research framework, we constructed a questionnaire “Research perspectives for drug development targeting parasitic vector-borne diseases and its environmental impact” (refer to underlying data: Supplementary File 1).

This questionnaire was disseminated online ( https://freeonlinesurveys.com), only to OneHealth *drugs* COST Action CA21111 collaborators. We took advantage of this interdisciplinary network, composed of a diverse group of researchers and stakeholders highly motivated and coordinated in the discovery and development of new environmentally friendly drugs effective against human and animal PVBDs. Besides the diversity of research backgrounds, this sampling also reflects range of research settings as research groups were based in 32 mostly European (n=28/32) countries ( Table 1).

**Table 1.** Geographical distribution of the OneHealth *drugs* inquired researchers and research groups.

Country of affiliation	Number of researchers	Number of research groups
Albania	2	2
Belgium	6	5
Bosnia and Herzegovina	2	2
Cameroon	1	1
Croatia	3	3
Cyprus	1	1
Czech Republic	2	1
Denmark	1	1
Finland	1	1
France	5	4
Germany	6	6
Greece	6	3
Iceland	1	1
Israel	3	2
Italy	21	6
Latvia	1	1
Lithuania	1	1
Macedonia	2	2
Malta	1	1
Poland	1	1
Portugal	7	4
Romania	2	2
Serbia	3	3
Slovakia	2	1
Slovenia	1	1
South Africa	1	1
Spain	4	3
Sweden	3	3
Switzerland	2	2
Tunisia	2	2
Turkey	7	7
UK	4	3
**TOTAL**	105	77

Responses were collected from March to December of 2023. The questionnaire consisted of 34 questions cataloguing the participating collaborators’ demographics and scientific expertise, as well as the composition and funding of the research groups (refer to underlying data: Supplementary File 1). Furthermore, the questions gauged the collaborators’ awareness of the environmental impacts of their work. From the 160 OneHealth *drugs* participating collaborators by December 2023 we collected 106 answers (response rate of 66%). The full survey data are available as Supplementary File 2 under underlying data.

All participating collaborators were full members of COST Action 21111 OneHealth *drugs* and made aware of the purpose and nature of the perceptions-knowledge-attitudes survey through meetings and emails. The survey preamble consisted of the statement given in the section **Ethical approval and consent,** and participation implied no objection to the clearly stated purpose.

### Survey findings

***Composition and funding of research groups.*** The survey provided a cross-sectional analysis of the research group demographics, funding resources, available technologies, and current research trends for PVBD drugs in Europe and neighbouring countries. Approximately 60% of the respondents are currently working on PVBD drug development. The majority of the inquired researchers work for Academia (87% vs 12% working for Governmental National Research Institutes, 4% working for the industry, 2% for private research foundations or institutes and 5% under other settings). Research groups mostly consist of a small number of researchers (63% with 1–4 researchers), very often at an early stage of career (63% below 35 years of age). Bachelor and Master students are overrepresented compared to PhD students (81% of the research groups have at least one Bachelor or a Master student, while 24% of the research groups do not include PhD students, and 68% of those who do, have only one to three PhD students) ( Table 2). Although this would appear to be an encouraging scenario for the future of PVBD drug research, it actually reflects a lack of funded PhD opportunities and scholarships in this field, causing research labs to be populated by less experienced researchers. The observation that most research teams consist of only 1–4 researchers may further suggest there is only limited financial support for this kind of research.

**Table 2.** Participation of Bachelor, Master and PhD students in OneHealth *drugs* associated research groups.

Number of students involved in drug development for PVBD	Bachelor and Master students	PhD students	Bachelor and Master students	PhD students	Bachelor and Master students	PhD students	Bachelor and Master students	PhD students	Bachelor and Master students	PhD students
	**0**	**0**	**1**	**1**	**2**	**2**	**3**	**3**	**>3**	**>3**
**% of OHD associated laboratories**	19	24	25	29	20	17	8	6	3	24

**Figure 1.**
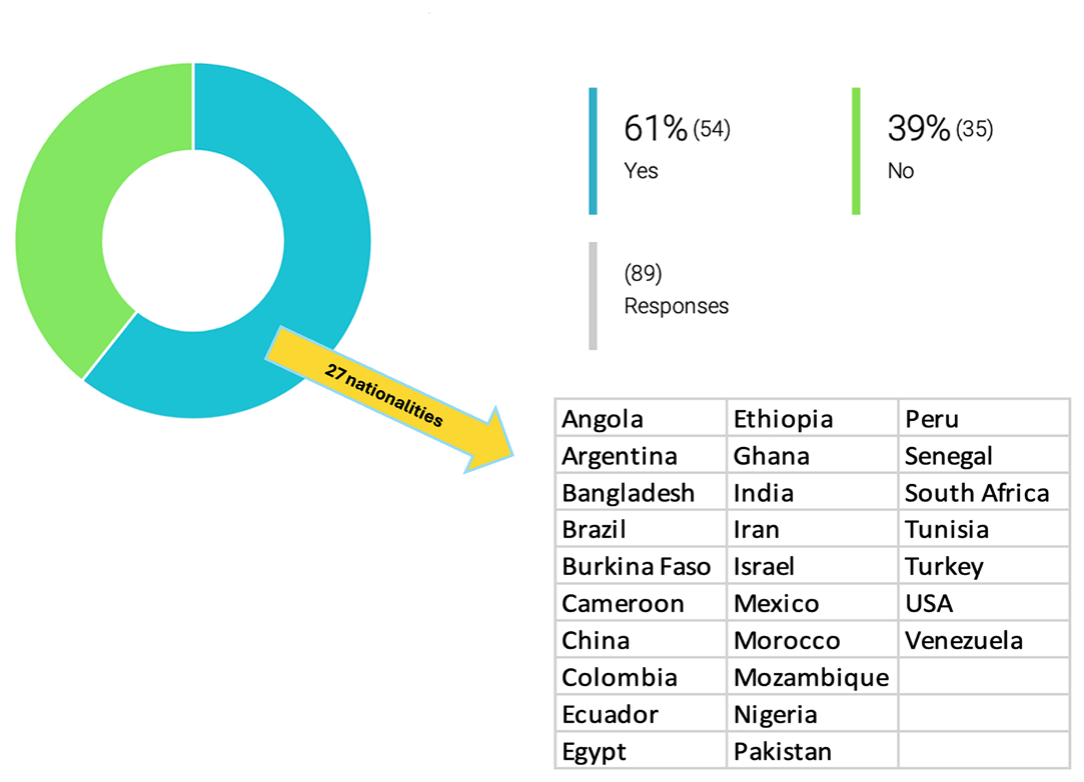
Involvement of non-European researchers in drug development for PVBD by the OneHealth *drugs* associated research groups. At least 61% (54/89) of the inquired researchers had at least one non-European colleague involved in projects on drug development for PVBDs. Among these, the following nationalities were described: India, Poland, Cameroon, Bangladesh, Egypt, Brazil, Sudan, Colombia, Nigeria, Kenya, China, USA, Argentina, Peru, Mexico, Venezuela, Canada, Nigeria, Bangladesh, Senegal, Morocco, Iraq, Ghana, and Israel.

The results of the survey furthermore indicate that the majority of the respondents are open to integrating international collaborators into their activities, with more than 60% of these research groups having already collaborated with non-European researchers ( Figure 1). This openness to collaboration is likely, at least in part, driven by the small group sizes and limited funding. On the other hand, few researchers have collaborated with governmental and/or private institutions dedicated to this scientific field (17% have worked with governmental institutions, 12% have worked with private institutions and 26% with both), presumably because few such opportunities present themselves.

The bulk of the research into PVBD drugs is being produced at the academic level, supported predominantly by national government and academic funding (as reported by 69% and 57% of the participating collaborators, respectively). Fewer researchers have taken advantage of funding from the European Commission or the private sector (25% and 28% of the respondents, respectively). PVBD-specific calls from the European Commission are few and even then, often limited to one specific disease like malaria.

**Figure 2.**
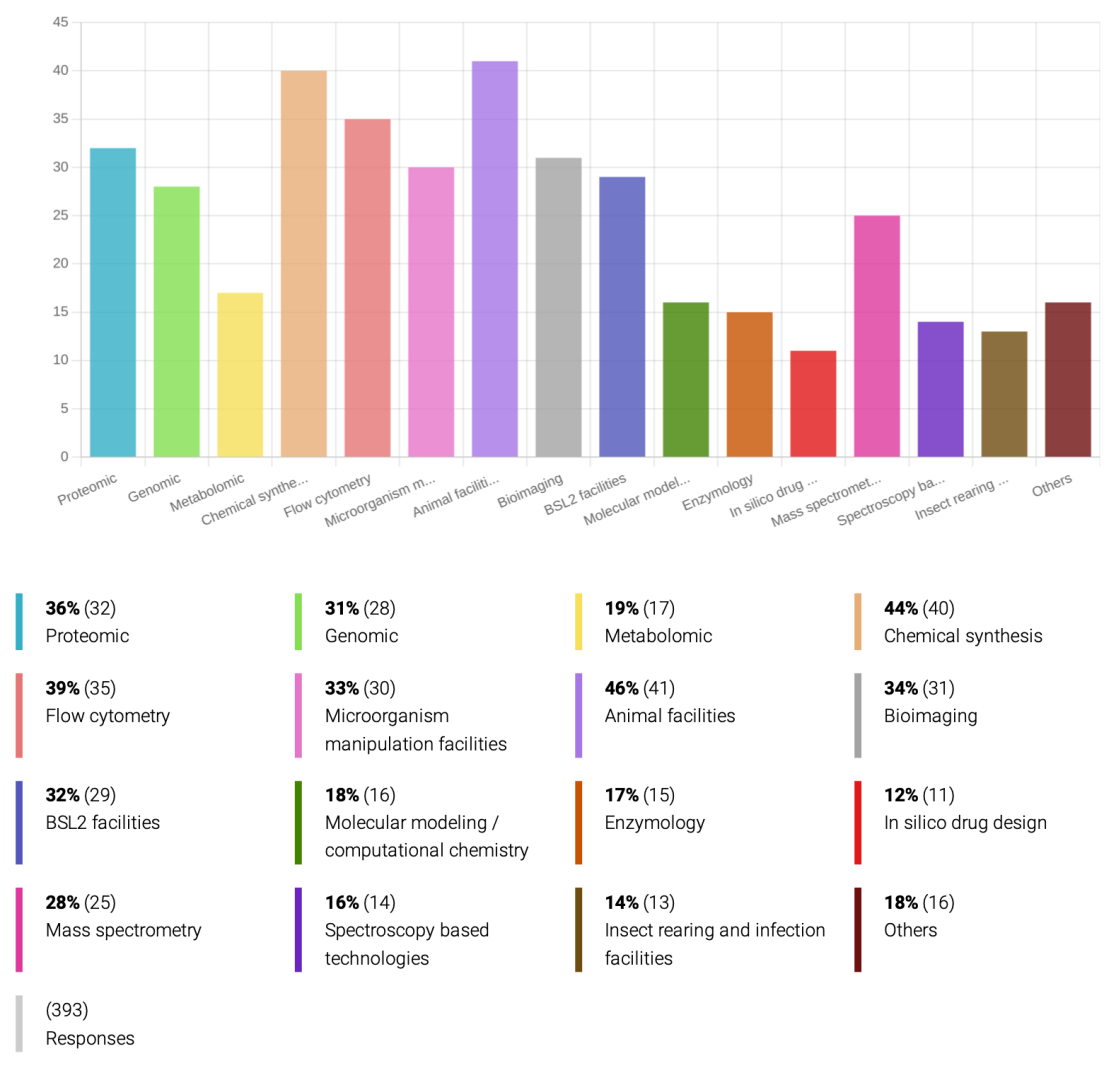
Technologies and materials employed by the inquired researchers on drug development for PVBD.

**Figure 3.**
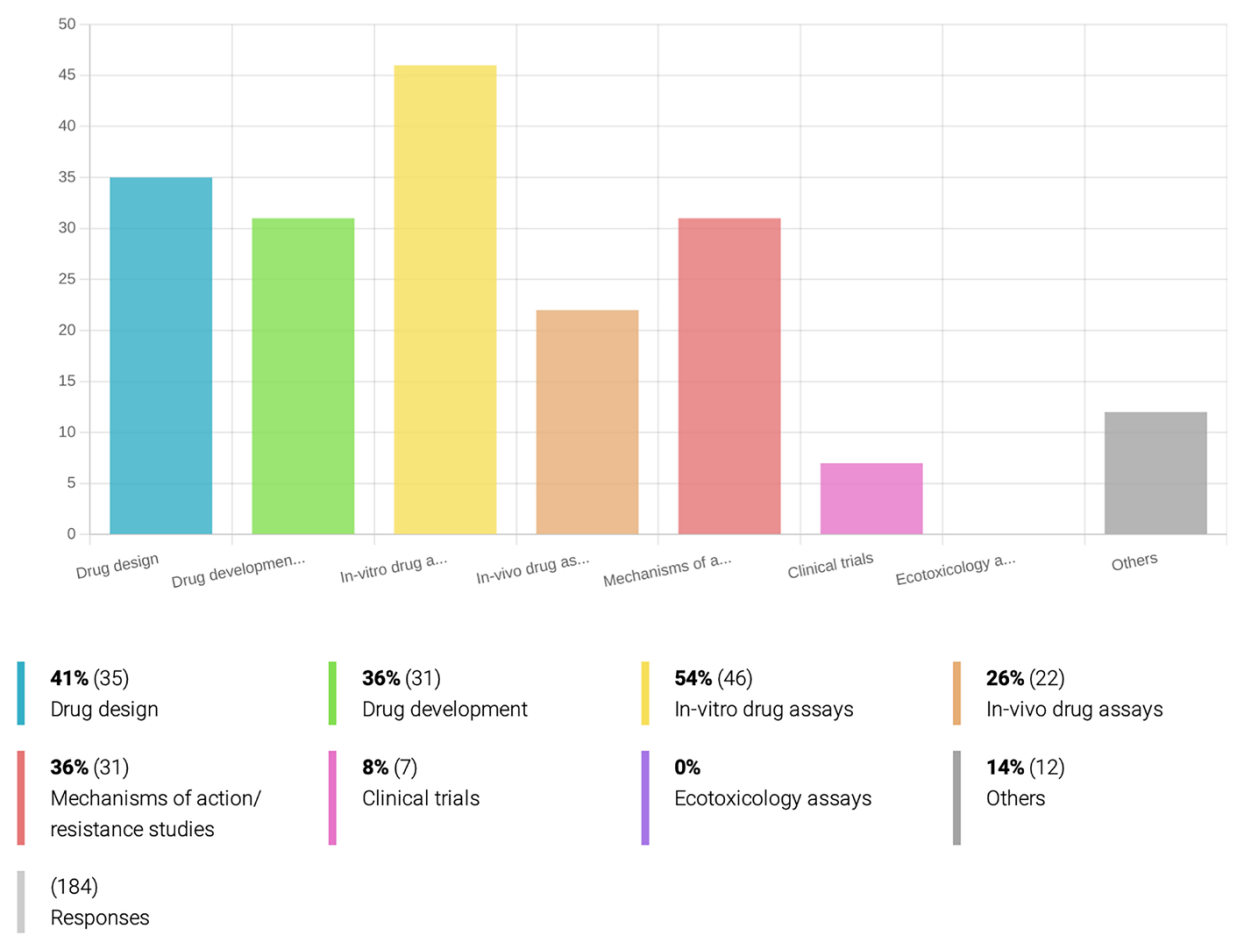
Research fields applied to drug discovery and development for PVBD. Among the inquired OneHealth *drugs* associated researchers who are currently involved in drug development for PVBDs, the majority (54%) works on *in-vitro* drug assays, drug design (41%), drug development (36%) and to the study of the compound’s mechanisms of action and resistance. A minority (8%) of the researchers is working on clinical trials, and none of the respondents is currently dedicated to ecotoxicology assays.

***PVBDs targeted and technologies and materials employed in R&D of new drugs.*** Respondents are particularly interested in drug development for protozoan-related diseases, such as leishmaniasis, malaria, Chagas disease, and African trypanosomiasis ( Table 3). Other parasitic vector-borne agents are underrepresented, especially in the helminth category but also in the tick-borne group, although drug discovery against those pathogens is just as urgent as for *Plasmodium* and the kinetoplastid protozoa. An important explanation behind this fact is how easy it is to culture and genetically manipulate these pathogenic protozoa, besides the existence of well-established models of infection, compared to almost all medically and veterinary relevant helminths and ectoparasites. Indeed, 33% of the inquired researchers have access to facilities for genetic manipulation of microorganisms ( Figure 2), and 54% of the respondents mentioned are involved with *in-vitro* drug sensitivity assays ( Figure 3), while only 14% have laboratory conditions for rearing and infecting insect vectors. At least 46% of the inquired researchers have access to animal facilities for *in vivo* studies ( Figure 2), but only 26% actually work on *in-vivo* drug assays ( Figure 3).

**Table 3.** Parasitic species targeted on drug developed projects by OneHealth *drugs* inquired researchers.

Parasite family	Parasite genus	Parasite species	Number of dedicated researchers	%
Protozoan	Babesia	*Babesia bigemina*	1	1.2
		*Babesia bovis*	2	2.3
		*Babesia divergens*	1	1.2
		*Babesia microti*	1	1.2
		*Babesia sp.*	1	1.2
	Besnoitia	*Besnoitia besnoiti*	1	1.2
	Leishmania	*Leishmania* sp.	4	4.62
		*Leishmania aethiopica*	1	1.2
		*Leishmania braziliensis*	3	3.6
		*Leishmania donovani*	3	3.6
		*Leishmania infantum*	21	24.1
		*Leishmania major*	2	2.3
		*Leishmania mexicana*	1	1.2
	Plasmodium	*Plasmodium berghei*	1	1.2
		*Plasmodium falciparum*	21	24.1
	Theileria	*Theileria annulata*	1	1.2
		*Theileria parva*	1	1.2
	Trypanosoma	*Trypanosoma sp.*	3	3.6
		*Trypanosoma cruzi*	4	4.60
		*Trypanosoma brucei*	8	9.2
		*Trypanosoma congolense*	1	1.2
Helminths	Schistosoma	*Schistosoma mansoni*	1	1.2
	Dirofilaria	*Dirofilaria immitis*	4	4.6
Total			87	100

**Figure 4.**
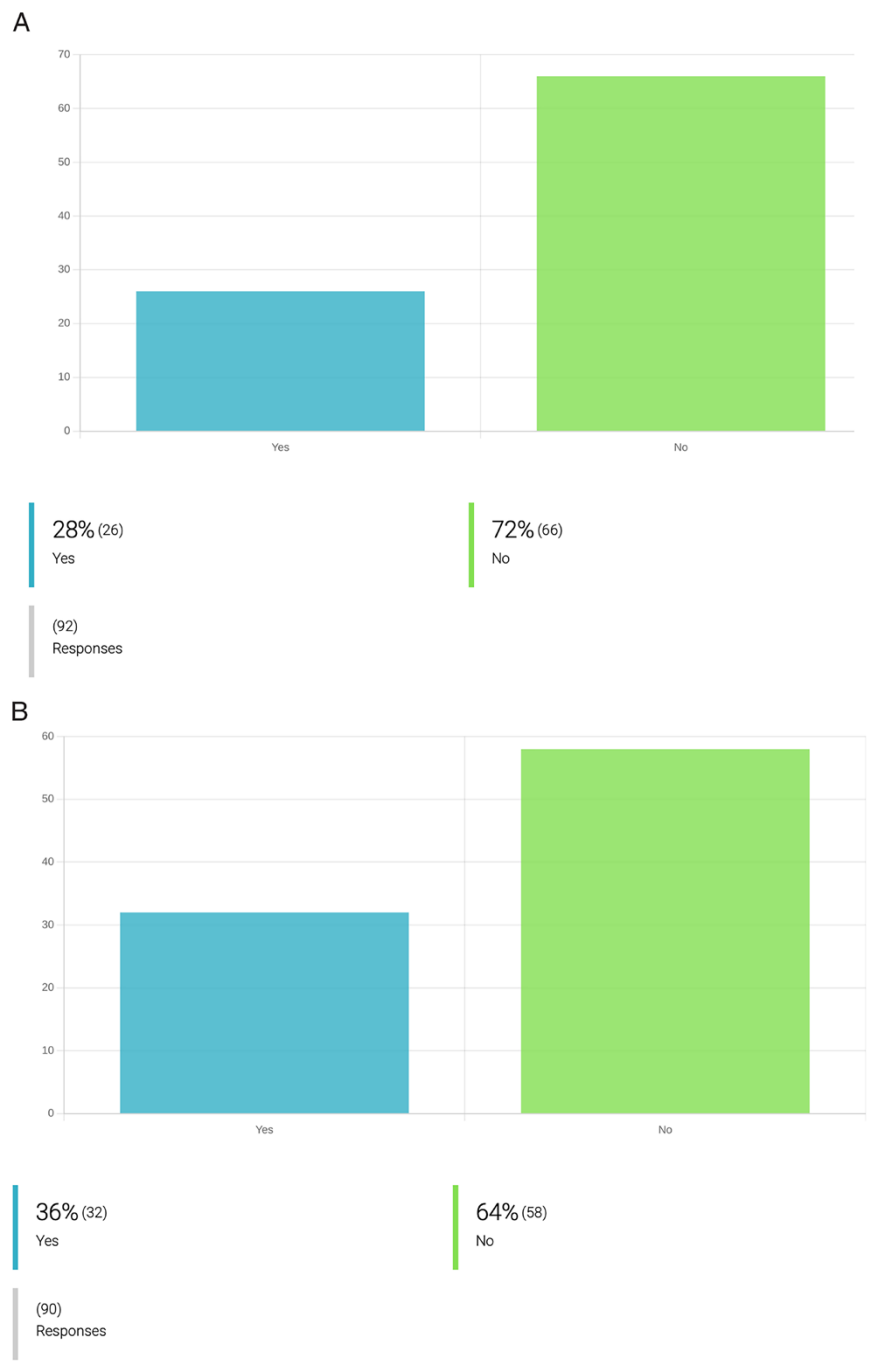
Attitudes and practices to mitigate water, energy and plastic consumption during drug development for PVBD. **A**. Incorporation of measures to reduce plastic use during the process of drug development against PVBDs. **B**. Incorporation of measures to reduce energy consumption during the process of drug development against PVBDs. Only 28% of the inquired researchers employ strategies to reduce plastic use during their research activities. These include: recycling, replacement of plastic materials by glass (ex. glass pipettes, glass wire; glass TLC plates; glass tubes); reduce the use of single plastic use equipment; optimize experiments to reduce waste production and plastic consumption (e.g. optimize the use of 96-well plates to fill all spaces available); recycle solvents; clean and re-use plastic for enzyme kinetics assays; replace plastic spectroscopic cuvettes by glass cuvettes. Regarding measures to reduce electricity consumption, only 36% of the inquired researchers employ at least one measure. These include: ultra-sound assisted synthesis, microwave-assisted synthesis (ex. MAOS); multicomponent reactions; limiting unnecessary illumination outside the normal working hours (e.g. lights off when the room is not used); limiting unnecessary heating outside the normal working hours; privileging the use of instruments with low energy consumption; routine inspection and maintenance of freezers (-20°C and -80°C) to avoid frost); selection of chemical synthesis protocols that involve milder conditions, with less energy consumption; implementation of institutional attitudes and practices that allow energy saving (e.g. new architectural designs of research buildings with energy saving systems); reducing the number of equipment kept on standby for long time; disconnect appliances and lab instruments when not used.

***Sustainable research and development practices.*** Surprisingly, the respondents showed only low awareness and motivation to implement strategies to reduce plastic, water and energy consumption, and increase the sustainability of their research practices ( Figure 4). This may, in part, be because of a lack of safe and cost-effective alternatives. For instance, governmental regulations requiring the incineration of consumables for pathogen cultures will not allow a change-over to glassware as a more sustainable option to plastic recipients, as this would bring added hazards of spills and accidental worker infection through breakage. Still, we argue there is scope for raising awareness and initiatives to make PVBD research more environment-conscious. There was, however, far more progress implementing the 3Rs principles in laboratory animal use. Only 22 out of the 105 inquired (21%) researchers responded that they use *in vivo* (animal) models in research for new drugs against PVBDs ( Figure 3). Twenty of these (90%) entered ‘yes’ when asked if they apply strategies to reduce such work. Moreover, an additional 31 researchers that had responded ‘no’ to the use of animals, indicated that they implemented 3R strategies – apparently to the extent of phasing out *in vivo* research altogether.

**Figure 5.**
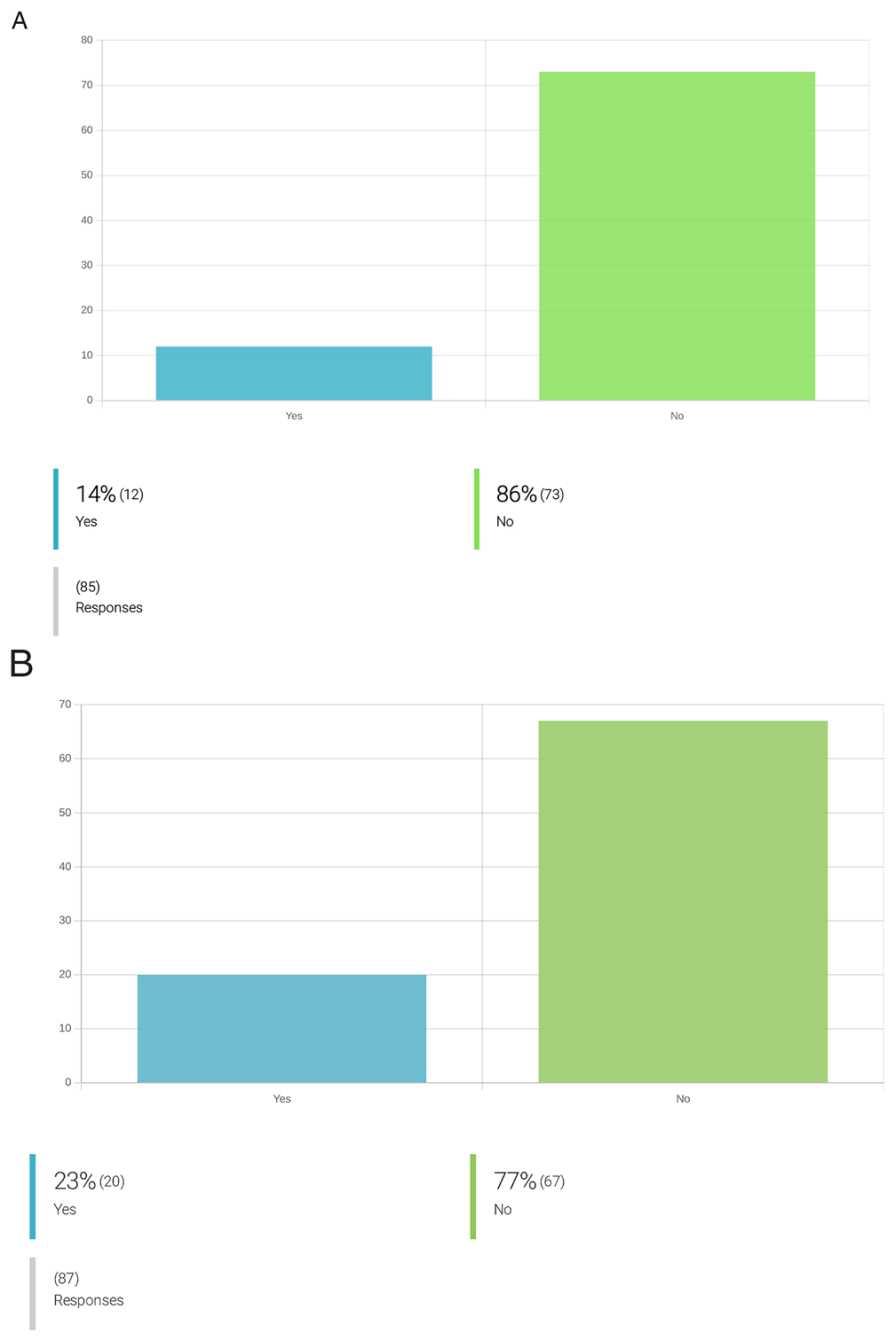
Attitudes towards integration of ecotoxicity and biodegradability assays in newly discovered lead compounds against PVBD. **A**. Integration of ecotoxicity assays for new compounds during drug development for PVBDs. **B**. Integration of biodegradability assays for new compounds during drug development for PVBDs. From the inquired researchers working on drug development for PVBDs, only 14% of the respondents claimed to include ecotoxicity prediction assays in the drug discovery pipeline. The most adopted organisms and models to address ecotoxicity include: testing ecotoxicity towards *C. elegans*, soil organisms, grass, mammalian cells and free-living protists. Regarding biodegradability assessment of a lead compound, 23% of the inquired respondents (n=20/87) already incorporate such assays in the drug discovery pipeline. For instance, a compound’s biodegradability is addressed by some researchers by selecting plant-derived compounds and biocompatible components; introducing functional groups that favour biodegradation; performing in-silico assays for the prediction of biodegradability, further using this information to prioritize target compounds; identifying drug metabolites under biometric conditions; performing *in-vitro* ADMET studies and exploring compounds previously synthesized by living organism; privileging compounds without halogen that can be degraded to just CO _2_ and water, and eventually ammonia, if they contain nitrogen.

**Figure 6.**
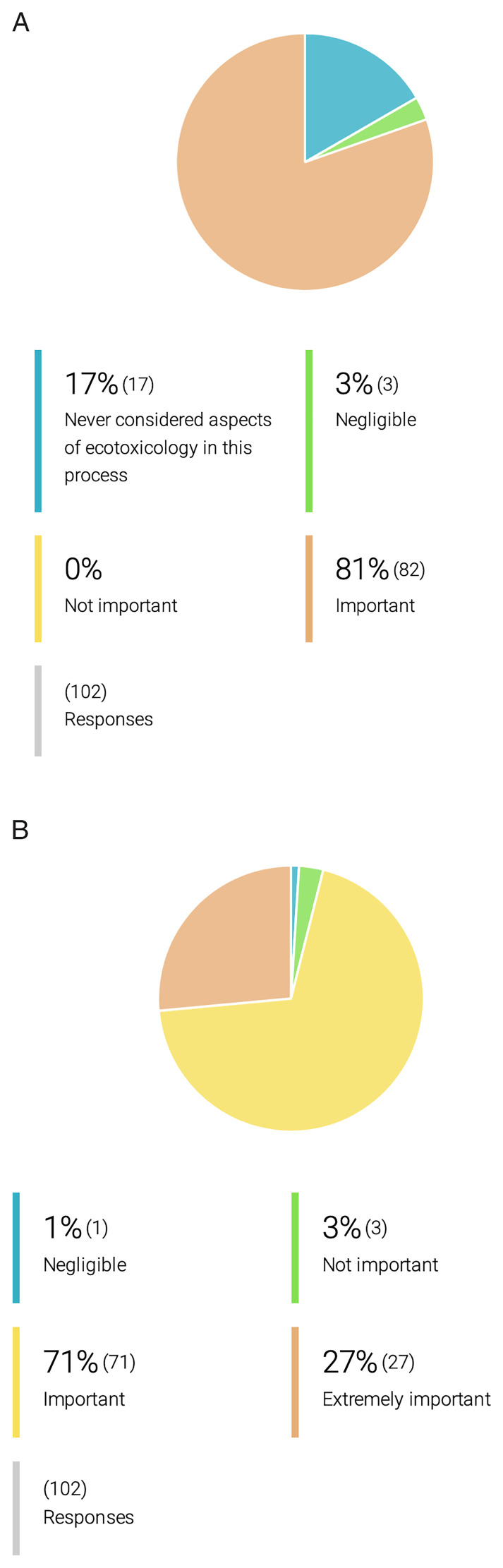
OneHealth *drugs* researcher´s opinion on the importance of ecotoxicological studies for drug development and marketing authorization. **A.** Researchers’ opinion on the importance of ecotoxicology studies in the process of drug development for PVBDs. **B**. Researchers’ opinion on the importance of incorporation of ecotoxicology studies in the marketing authorization for drugs against PVBDs.

***Implementation of ecotoxicology goals.*** At the onset of the OneHealth *drugs* COST Action, only 14% of its collaborators considered aspects of ecotoxicology during the early stages of their research, and only 23% reported addressing biodegradability aspects during the discovery process of parasiticidal compounds ( Figure 5). Yet, 91% of the researchers who do not implement biodegradability or ecotoxicology studies during their research, support their inclusion. Indeed, 71% of respondents support even the inclusion of ecotoxicological assessments in the application of marketing authorization for a new drug ( Figure 6).

From our survey, it appears that the majority of respondents are unfamiliar with ecotoxicological test approaches and/or lack the necessary expertise and resources for integrating such approaches into their research. Therefore, it seems necessary to invest in an improved understanding and assessment of drugs’ biodegradability in the environment and their ecotoxicity, including appropriate workflows to include these topics in PVBD drugs research. This will require the creation of appropriate training courses for the researchers. Relevant, reliable, and standardized protocols are urgently needed to allow robust, systematic and reproducible assessments of the environmental risks of new drug candidates. For example, in order to keep initial costs to a level that most participating PVBD groups can afford, a limited set of standard tests should be agreed upon as a go/no-go decision point. Moreover, a set of general guidelines with rule-of-thumb indicators of compound characteristics likely to cause or avoid ecotoxicity will inform the synthesis strategies of the participating chemical laboratories.

Collaborations between individual groups dedicated to different but complementary activities promotes capacity building. This is particularly relevant from the ecotox assessment point of view, where partnerships between researchers in the field of medicinal chemistry, pre-clinical trials and ecotoxicology can fill in gaps of knowledge and implement best recommendation practices to achieve a green transition.

## Discussion

### Potential interventions to mitigate environmental impact of new drugs for PVBD

Among other factors, the success of global *One Health* strategies relies on biomedical innovation. Within this field of research, drug development plays a pivotal role in the fight against many of the infections that plague humans and animals. However, the progress of biomedical research and innovation is costly, lengthy, and depends not only on highly educated, trained, and specialized workers, but also places a high demand on power and other carbon resources. The contribution to the production of global waste from biomedical research is not insignificant, as it depends largely on chemical reagents and solvents, single-use plastic consumables and electronic equipment, among others, that result in large amounts of solid waste, biological waste, wastewater, pollutants, and energy consumption, increasing the pressure on an already damaged climate and polluted natural world. In addition to the direct environmental impact of biomedical research, we must also account for the potential ecological damage produced by many of the biomedical research end products, namely drugs and other medical devices.

Parasiticides are essential drugs for both human and animal health. Like antibiotics, parasiticides have become essential in livestock rearing and food security. It is important that new drugs have a minimal environmental impact, at all stages of their life cycle. However, the level of ecological risk of the current pharmacopeia for PVBD is almost completely unknown: we often do not even know the drug’s metabolites and “end-of-life” residues. Nor can the current drugs be easily discontinued if judged to have undesirable ecological impacts, as there is little redundancy in the pharmacological armoury against PVBDs.

This is not to argue that the environmental impact of the current antiparasite drugs should not be evaluated – indeed, ecopharmacovigilance is essential to prevent the disastrous consequences of causing severe ecological upsets ^39^, such as illustrated by the example of diclofenac mentioned above. For the current period, then, where new, safer drugs for most PVBDs are not on the horizon, environmental safety goals must continue to be balanced against the need for treatment, including mass administrations and prophylaxis to probably healthy patients or animals – but they must be based on sound ecotoxicological assessments and monitoring. Including such assessments into the drug development pipeline will allow us to prioritize the replacement of those drugs that are considered to have the largest negative environmental impact, and, crucially, include that evidence in the case to relevant funders. In parallel, the environmental impact of the research process should also be carefully and continuously monitored. Research should operate at minimal carbon waste, minimal chemical pollution, maximum reduction, recycling, re-use and repurpose, while favouring state of the art alternatives for animal models during both preclinical research and clinical trials.

While the results of our survey offer valuable insights into how a group of dedicated researchers in the field of drug development for PVBD perceive and potentially deal with the environmental impact of their work, we here highlight and reflect on some of the analysed topics, suggesting possible ways of coping with such challenges going forward.

### Recommendations

Regarding possible interventions to “green-up” the drug development pipeline and mitigate its environmental impact, recommendations were stratified in two categories of applicability: “ready to put in place” and “continuous effort”.

In the “ready to put in place” category, we include general considerations for research sustainability, technology companies, and human resources ( Table 4). These considerations rely mostly on the 3R principles for waste management (reduce, re-use, and recycle) and for animal experimentation (replacement, reduction, and refinement), as well as new considerations to improve the sustainability of the current pipeline of drug discovery. In the “continuous effort” category we include aspects to be considered by different stakeholders, including funding agencies and regulatory bodies involved in drug development research ( Table 5).

**Table 4.** “Ready to put in place” measures to reduce carbon-associated emissions during drug development for PVBD.

Required attitudes and behaviour change	Prompt benefits
Reduce single-use plastic.	Cost saving, decrease carbon footprint, decrease plastic waste production, healthier environment.
Reduce energy consumption.	Cost saving, decrease carbon footprint, healthier environment.
Replace, reduce, and refine use of laboratory animals.	Protect animal welfare, save on cost, decrease carbon footprint, healthier environment.
Include education towards *One Health,* Planetary Health and Principles of Sustainability and good management of natural resources into Pharmacology, Biology and MedChem curriculae.	Ground the future generations of researchers in sustainable laboratory practices for solid and long-lasting transformation into greener research practices and environmentally safer drugs.
Establish models to assess the ecotoxicological impact of drugs for PVBDs and incorporate such evaluation into the drug development pipeline.	Early removal of any drug with severe ecotoxicological impact from the drug discovery pipeline; balanced decision making by weighing the environmental burden, *in its intended ecological setting* ^1^, against the pharmacological advance for a neglected PVBDs.
Incorporate the assessment of a new compound’s biodegradability and ecotoxicological effects as a pre-requisite for a marketing authorization.	Avoid introducing drugs with potentially severely negative expected impact on the environment to the market.

^1^e.g. marine ecotoxicity is highly relevant for a drug used in fish farming but less so for a drug intended to treat trypanosomiasis in camels.

**Table 5.** Recommendations for stakeholders directly involved in PVBD drug development, manufacturing or prescription.

Recommendations for stakeholders	Prompt benefits
To urge relevant funding agencies to support investment in research sustainability by promoting the exploitation of research sustainability best practice, multidisciplinary collaborations and the development of guidelines.	Financially and logistically support for research institutions in their efforts to reduce the environmental impact of their research activities.
	Encourage researchers to improve their capacity building by learning from others and incorporating aspects of research sustainability in their daily work.
	Guidelines to support researchers to develop and incorporate pro-sustainability strategies in their project development, grant applications and future research programmes.
To encourage government and EU funding agencies to apply specific criteria for research sustainability strategies and practices in the evaluation of grant applications, following the example of the 3Rs in laboratory animal usage.	Encorage the implementation of green research practices and research sustainability in all projects.
To raise awareness and education among health providers in both human and veterinary fields to adopt safe and evidence-based prescription practices and sound strategies to minimize the impact of drug waste, over-use, and the concentration of drug (metabolites) in the environment.	Avoid drug waste, minimize prescription/ therapy associated costs, reduce the introducing of drugs with potentially negative impact on the environment.
To investigate the ecotoxicological impact of the antiparasitic drugs currently available, licensed and prescribed for prevention and treatment of PVBD, and develop proposals on how to minimize such impacts.	Allow the prioritization of the replacement of the drugs considered to have the largest negative environmental impact and avoid repurposing such compounds for additional uses.
To introduce funding mechanisms for joint academic/private sector for the development of new treatments to replace the current antiparasitic drugs with the worst environmental footprint.	Bridging Academia and industry should accelerate the process of drug development and manufacturing.

Environmental sustainability of research should be integrated as a fundamental effort for better research practices. Sustainability assessment should be considered prior the execution of each drug development process. To achieve this, universal criteria for assessment of sustainable research should be made available through standardized operating procedures (SOPs), guidelines, and frameworks. These would likely facilitate capacity building and the training of researchers, laboratory technicians, and support staff. Agreed tests would allow the development of standard-setting scales for environmental impact that allow objective decisions towards development and use of antiparasite drugs.

We therefore propose the development of an independent consultancy agency that, based on scientific evidence, can offer training and access to information that supports good laboratory practices for sustainable research in drug development, including for PVBD. This entity should have the capacity to provide guidance to institutions and research groups/laboratories on how to macro and micro-manage the available resources and invest in sustainable research infrastructures, equipment, and practices. In addition, based on standardized metrics, such an agency could provide tools and training on monitoring the environmental impact of a research institute or laboratory, evaluate its carbon footprint progression, while providing support in decision-making to reduce its impact as needed.

Table 6 lists several further recommendations for policy makers. These include the encouragement of sustainable practice by laboratory suppliers, increased data collection and openness on the impacts of antiparasite drug production, distribution and use, and increased monitoring of the environmental impacts of antiparasite drugs. The creation of momentum towards greener priorities for research will have the added important effect of incentivising suppliers to meet that demand with product innovation.

**Table 6.** Recommendations for policy makers.

Recommendations for policy makers	Prompt benefits
To expand the application of sustainable practices to all contributors to the supply chain of research materials and goods (e.g., transporting, packaging, producing, distributing).	Globalization of norms to favour good practices in research for PVBD of humans and animals. Comply with the United Nations Sustainable Development Goals. Comply with the European Union Green Deal. Promote One Health for all. Promote science-driven regulations.
To require data sharing across sectors by increasing transparency and open access to information on the production and distribution of antiparasitic drugs of human and animal use (industry production, distribution, prescription).	
Set up regular and compulsory control programmes for monitoring the environmental impact of drugs for PVBD in the water and soil, and their safety for vegetation, microbes and aquatic organisms.	

## Conclusions

By surveying collaborators of the OneHealth *drugs* COST Action, a consortium of researchers based across Europe and neighbour countries dedicated to the discovery and development of drugs for PVBD of humans and animals, we were able to collect important first insights into the general research structure and directions of ongoing drug discovery against PVBD, and how this community endeavours to develop effective parasiticidal drugs that are safe for the environment. Specifically, most groups are small and rely on early-career researchers, with many groups having more undergraduate and Master-level students than more experienced workers, likely reflecting a sparsity of available research funds in this research field. Awareness of environmental issues and the need for increased sustainability in the research is high, but few researchers felt able to change their impact substantially, highlighting the need for the sharing of ideas, information on greener products and best practice. The creation of an advisory body could play an important role in advancing these ambitions.

As with sustainable research and development, the survey found an almost unanimous agreement that ecological evaluation should be part of drug development, although this is not yet common practice today: there is a paucity of know-how, as well as a lack of capacity and training in this area. Training schools would help address this deficiency, as would proposals for the standardisation of protocols and tests, with agreed scales allowing rational go / no-go decision making early in the drug discovery pipeline. Guidelines should be drawn up to help identifying chemical classes with expected high environmental impact that should ideally be avoided, thereby reducing dead-ending projects, saving time and resources, and reducing the overall environmental impact of drug discovery. One such class of persistent and toxic compounds are the perfluoroalkyl and polyfluoroalkyl substances (PFAS) ^57^, commonly known as “forever chemicals” which are often found in agricultural products such as various pesticides ^58^.

In parallel, funding should be sought to systematically assess the ecological impact of the current anti-PVBD drugs in use. This is essential in order to make rational decisions about the prioritisation of drug replacements and phase-outs. It must be remembered that the environmental impact of a drug may be in its production as well as its use. One example that comes to mind is the continued use of heavy metal-based drugs, such as arsenic-based compounds against both human and animal trypanosomiasis (melarsoprol and melarsomine, respectively). Similarly, antimony-based formulations remain the mainstay of leishmaniasis treatment in most endemic countries, although liposomal amphotericin B and miltefosine are available alternatives ^59^. However, while one may expect that the heavy metal-based drugs are particularly damaging to the environment (production and usage), to the best of our knowledge the environmental impact of none of these drugs has been comprehensively investigated, and certainly no standardized criteria to allow rational evaluation of their relative ecotoxicity have been produced.

In summary, a comprehensive research programme into the environmental risks of antiparasite drugs is long overdue and must be incorporated into the management of PVBDs and the ongoing efforts towards new treatments. Similar efforts are underway regarding the impact of insecticides, herbicides, antibiotics and cancer drugs. It is imperative that the antiparasitic drug community engages with those efforts and incorporates appropriate standards into their drug development pipeline, not only because this is the right thing to do but also because regulation promises to become more stringent—ready or not.

## Ethical approval and consent

Ethical issues were evaluated by the Ethics Committee at the University of Modena, Italy, as U-Modena are the grant holders for the COST Action CA21111 OneHealth *drugs* under which the suervey was conducted, but no formal approval was required, as all collaborators were full members of CA21111 and made aware of the purpose and nature of the perceptions-knowledge-attitudes survey through meetings and emails. The survey preamble consisted of the following statement and participation implied no objection to the clearly stated purpose:

*“This questionnaire is being conducted under COST Action 21111 on “One Health drugs for Vector-Borne Diseases”, aiming to survey the current trends and status of the research and drug development for the treatment of Vector-Borne Diseases. In addition, we intend to assess the level of awareness about the sustainability and environmental impact of the process of developing drugs for parasitic Vector-Borne Diseases (PVBDs). To answer each question, please select one or more options to reply to each question. The questionnaire is composed of 33 questions and shouldn't take more than 20 min to reply. The answers will be summarised and analysed. From the results of this survey, we aim to develop training opportunities and guidelines to help researchers and their institutes produce more sustainable and environmentally safe compounds for the treatment of Parasitic Vector-Borne diseases.”*

The Ethics Committee therefore waived the need for obtaining any further form of consent for the participating collaborators.

## Data Availability

BioStudies: Underlying for ‘Environmental impacts of drugs against parasitic vector-borne diseases and the need to integrate sustainability into their development and use’. This project contains the following data: Supplementary File 1: "Data collection on research perspectives for drug development targeting vectorborne diseases and environmental impact"; BioStudies accession number S-BSST1447, https://www.doi.org/10.6019/S-BSST1447 ^60^. Supplementary File 2: “Survey returns from survey on research perspectives for drug development targeting vectorborne diseases and environmental impact”. Survey results by participating collaborator. BioStudies accession number S-BSST1509, https://www.doi.org/10.6019/S-BSST1509 ^61^. Data are available under the terms of the Creative Commons Zero “No rights reserved” data waiver (CC0 1.0 Public domain dedication).
