# Supplementary material for: Environmental impacts of drugs against parasitic vector-borne diseases and the need to integrate sustainability into their development and use
Source: Open Res Eur. 2024 Nov 4;4:207. Originally published 2024 Sep 23. [Version 2] doi: 10.12688/openreseurope.18008.2 (PMC11555358; doi:10.12688/openreseurope.18008.2)
Supplement: Peer review history

# Reviewer report: Reviewer response for version 2

**DOI:** 10.21956/openreseurope.20400.r45819

**Published:** 2024-11-11

**Authors:** Unax Lertxundi

**Affiliations:** 1 Bioaraba Health Research Institute, Vitoria-Gasteiz, Spain

The authors have adequetely addressed my comments.

Is the study design appropriate and does the work have academic merit?

Yes

Is the work clearly and accurately presented and does it cite the current literature?

Partly

If applicable, is the statistical analysis and its interpretation appropriate?

Not applicable

Are all the source data underlying the results available to ensure full reproducibility?

Yes

Are the conclusions drawn adequately supported by the results?

Yes

Are sufficient details of methods and analysis provided to allow replication by others?

Partly

Reviewer Expertise:

Pharmaceuticals in the environment. Pharmacovigilance. Psychopharmacology

I confirm that I have read this submission and believe that I have an appropriate level of expertise to confirm that it is of an acceptable scientific standard.

# Reviewer report: Reviewer response for version 2

**DOI:** 10.21956/openreseurope.20400.r45818

**Published:** 2024-11-11

**Authors:** Idris Olatunji Sanusi

**Affiliations:** 1 Pharmaceutical Chemistry and Analysis, Kampala International University, Kampala, Central Region, Uganda

The authors have now satisfactorily addressed all my comments and also improved the quality of the manuscript. Therefore, I recommend acceptance of the research article.

Is the study design appropriate and does the work have academic merit?

Yes

Is the work clearly and accurately presented and does it cite the current literature?

Yes

If applicable, is the statistical analysis and its interpretation appropriate?

Partly

Are all the source data underlying the results available to ensure full reproducibility?

Yes

Are the conclusions drawn adequately supported by the results?

Yes

Are sufficient details of methods and analysis provided to allow replication by others?

Partly

Reviewer Expertise:

Environmental Chemistry; Medicinal Chemistry; Drug Development and Delivery

I confirm that I have read this submission and believe that I have an appropriate level of expertise to confirm that it is of an acceptable scientific standard.

# Reviewer report: Reviewer response for version 1

**DOI:** 10.21956/openreseurope.19463.r44637

**Published:** 2024-10-09

**Authors:** Unax Lertxundi

**Affiliations:** 1 Bioaraba Health Research Institute, Vitoria-Gasteiz, Spain

The authors have performed a survey about the general research structure and directions of ongoing drug discovery against human and animal parasitic vector-borne diseases (PVBD) in the context of the COST Action 21111- “One Health drugs”. The manuscript is well written, and presented data are of great interest.

 However, I believe the title of the manuscript does not adequately reflect its contents. It should acknowledge that the manuscript describes and discusses the results of a survey. The abstract section also needs some improvement. The total number of surveyed individuals should be clearly detailed here. Methods section in the abstract should define that the survey was disseminated only to COST Action CA21111 collaborators.

 The article contains 7 figures and 6 tables. Maybe some of them might be added as supplementary material?

 Additional comments

- In line of the COST Action “OneHealthDrugs” other authors ^1^ have suggested applying the One Health philosophy to other therapeutic groups apart from antibiotics. The rabies cases after the vulture population collapse in India-Pakistan is a good example of that ^2^.
- Pag 5, maybe a mention to the effects of ivermectin on terrestrial organisms could be added. Ivermectin has been shown to bioaccumulate in dung beetles too ^3^ .
- The authors may find this study about malaria and vector control with invermectin interesting ^4^.
- This letter published in Lancet about mass drug administration programmes may result interesting too ^5^.
- On pag 13, “ *Nor the current drugs be easily discontinued if judged to have underisable ecological impacts*”. This is an important statement. I agree. An adequate framework to assess environmental risks is currently lacking. In other words, we need to do ECOPHARMACOVIGILANCE. Maybe a mention about how a veterinary pharmaceutical (diclofenac) was withdrawn from India, Pakistan and Bangladesh due to its impact on vulture population.
- On pag 15, “ *guidelines should be drawn up to help identifying chemical classes with expected high environmental impact*”. Maybe a mention to PFAS?  As suggested in ^6^ .
- More information about PFAS and parasitic diseases: many pesticides used for vector control are PFAS: https://www.lrl.mn.gov/docs/2024/mandated/240221.pdf
- Although not directly related to the issue of “drugs for the treatment of PVBDs, this manuscript about the effects of other pharmaceuticals such as SSRI on schistosomiasis vector snails ( *Biomphalaria*) may result interesting too ^7^ .
- Consider adding some mention to pet-pharmaceuticals? The number of pets is growing importantly in Europe ^8^ ; ^9^.

Is the study design appropriate and does the work have academic merit?

Yes

Is the work clearly and accurately presented and does it cite the current literature?

Partly

If applicable, is the statistical analysis and its interpretation appropriate?

Not applicable

Are all the source data underlying the results available to ensure full reproducibility?

Yes

Are the conclusions drawn adequately supported by the results?

Yes

Are sufficient details of methods and analysis provided to allow replication by others?

Partly

Reviewer Expertise:

gd

I confirm that I have read this submission and believe that I have an appropriate level of expertise to confirm that it is of an acceptable scientific standard, however I have significant reservations, as outlined above.

## References

[1] : Greening the pharmacy. *Science* .2022;377(6603) : 10.1126/science.abp9554 259-260 10.1126/science.abp9554 35857602

[2] : The Social Costs of Keystone Species Collapse: Evidence from the Decline of Vultures in India. *SSRN Electronic Journal* .2023; 10.2139/ssrn.4318579 10.2139/ssrn.4318579

[3] : Biomagnification and body distribution of ivermectin in dung beetles. *Sci Rep* .2020;10(1) : 10.1038/s41598-020-66063-0 9073 10.1038/s41598-020-66063-0 32493927 PMC7270108

[4] : An ecotoxicological view on malaria vector control with ivermectin-treated cattle. *Nature Sustainability* .2024;7(6) : 10.1038/s41893-024-01332-8 724-736 10.1038/s41893-024-01332-8

[5] : Mass drug administration: time to consider drug pollution?. *The Lancet* .2020;395(10230) : 10.1016/S0140-6736(20)30053-2 1112-1113 10.1016/S0140-6736(20)30053-2 32247389

[6] : GREENER Pharmaceuticals for More Sustainable Healthcare. *Environ Sci Technol Lett* .2022;9(9) : 10.1021/acs.estlett.2c00446 699-705 10.1021/acs.estlett.2c00446 36118957 PMC9476650

[7] : Environmentally relevant concentrations of the tricyclic antidepressant, amitriptyline, affect feeding and reproduction in a freshwater mollusc. *Ecotoxicol Environ Saf* .2024;281: 10.1016/j.ecoenv.2024.116656 116656 10.1016/j.ecoenv.2024.116656 38945099

[8] : Environmental contamination by pet pharmaceuticals: A hidden problem. *Sci Total Environ* .2021;788: 10.1016/j.scitotenv.2021.147827 147827 10.1016/j.scitotenv.2021.147827 34134354

[9] : Is it time to re-evaluate anti-parasitic use in companion animals?. *Companion Animal* .2024;29(3) : 10.12968/coan.2023.0067 12-18 10.12968/coan.2023.0067

# Reviewer report: Reviewer response for version 1

**DOI:** 10.21956/openreseurope.19463.r44634

**Published:** 2024-10-09

**Authors:** Idris Olatunji Sanusi

**Affiliations:** 1 Pharmaceutical Chemistry and Analysis, Kampala International University, Kampala, Central Region, Uganda

I have read and reviewed the manuscript titled "Environmental impacts of drugs against parasitic vector-borne diseases and the need to integrate sustainability into their development and use”.

 The research is well designed and the manuscript is well compiled and fit well to the scope of the Journal. I therefore recommend accept with minor correction. The minor comments are;

1. Provide reference for the second sentence under the "Introduction” section.
2. “Besides the diversity of research backgrounds, this sampling also reflects various research settings as research groups were based in 32 different countries, predominantly European (n=28) but also several others (n=4)”. Please revise the sentence.
3. “Responses were collected between March and December of 2023” Change to Responses were collected from March to December 2023.
4. The authors did not include analytical techniques employed for the detection of anti-parasitic drugs in environmental samples. If there is any information on the analytical techniques for detection, please provide.
5. Some sentences should be joined together to make one paragraph. There are several paragraphs under “Discussion” section, please revise the “discussion” aspect.
6. The recommendations are well organized and outlined. However, it should be included as a section (heading) in the manuscript before the conclusion.
7. The conclusion also needs to be revised.

Is the study design appropriate and does the work have academic merit?

Yes

Is the work clearly and accurately presented and does it cite the current literature?

Yes

If applicable, is the statistical analysis and its interpretation appropriate?

Partly

Are all the source data underlying the results available to ensure full reproducibility?

Yes

Are the conclusions drawn adequately supported by the results?

Yes

Are sufficient details of methods and analysis provided to allow replication by others?

Partly

Reviewer Expertise:

Environmental Chemistry; Medicinal Chemistry; Drug Development and Delivery

I confirm that I have read this submission and believe that I have an appropriate level of expertise to confirm that it is of an acceptable scientific standard.

# Response

**Published:** 2024-10-30

**Authors:** Harry De Koning

**Affiliations:** School of Infection and Immunity, University of Glasgow, Glasgow, Scotland, UK

Comment 1 response: We have added references to recent papers addressing the concept as animals as patient, and patient-focussed health care delivery in veterinary practice, and patient centered care (PCC) in human medicine. The discussion is an important one and PPC is not always axiomatic. However, in the treatment of parasitic diseases of man and animals, the case for treatment is far more straightforward than for many areas of medicine beyond infectious disease: one tries to eliminate the pathogen and that is in the best interest of the patient. Added references: Weich K, Grimm H: Meeting the patient’s interest in veterinary clinics. Ethical dimensions of the 21st century animal patient. Food Ethics. 2018; 1: 259-272. Springer S, Sandøe P, Bøker Lund T, et al.: “Patients’ interests first, but…”–Austrian veterinarians’ attitudes to moral challenges in modern small animal practice. Animals. 9(5): 241. Davis K, Schoenbaum SC, Audet AM: A 2020 vision of patient-centered primary care. J Gen Intern Med. 2005; 20(10): 953-957.

Comment 2 response; We have simplified the sentence to: “Besides the diversity of research backgrounds, this sampling also reflects a range of research settings as research groups were based in 32 mostly European (n=28/32) countries.”

Comment 3 response: The suggestion was implemented.

Comment 4 response: A paragraph has been added at the end of the section Environmental Impacts, including up-to-date references.

Comment 5 response: We agree that there were several single-sentence paragraphs in what is now the Recommendations section (see answer below, point 6), and that this is not good style. We have combined them into one more substantial paragraph and this makes the text more easily comprehensible.

Comment 6 response: Agreed and implemented.

Comment 7 response: We note that the reviewer did not indicate in what way the Conclusions section should be revised. We feel that appropriate and germane points were made. However, we have managed to shorten the section somewhat without losing any information.
